# Exploring Peritumoral Neural Tracts by Using Neurite Orientation Dispersion and Density Imaging

**DOI:** 10.3389/fnins.2021.702353

**Published:** 2021-09-27

**Authors:** Shin Tai Chong, Xinrui Liu, Hung-Wen Kao, Chien-Yuan Eddy Lin, Chih-Chin Heather Hsu, Yi-Chia Kung, Kuan-Tsen Kuo, Chu-Chung Huang, Chun-Yi Zac Lo, Yunqian Li, Gang Zhao, Ching-Po Lin

**Affiliations:** ^1^Institute of Neuroscience, National Yang Ming Chiao Tung University, Hsinchu, Taiwan; ^2^Department of Neurosurgery, First Hospital of Jilin University, Changchun, China; ^3^Department of Medical Imaging, Hualien Tzu Chi Hospital, Buddhist Tzu Chi Medical Foundation, Hualien, Taiwan; ^4^Department of Radiology, School of Medicine, Tzu Chi University, Hualien, Taiwan; ^5^GE Healthcare, Taipei, Taiwan; ^6^Center for Geriatrics and Gerontology, Taipei Veterans General Hospital, Taipei, Taiwan; ^7^School of Psychology and Cognitive Science, Institute of Cognitive Neuroscience, East China Normal University, Shanghai, China; ^8^Institute of Science and Technology for Brain-Inspired Intelligence, Fudan University, Shanghai, China; ^9^Brain Research Center, National Yang Ming Chiao Tung University, Hsinchu, Taiwan

**Keywords:** neurite orientation dispersion and density imaging, diffusion tensor imaging, fiber tractography, vasogenic edema, brain tumor, neurosurgery

## Abstract

Diffusion Tensor Imaging (DTI) tractography has been widely used in brain tumor surgery to ensure thorough resection and minimize functional damage. However, due to enhanced anisotropic uncertainty in the area with peritumoral edema, diffusion tractography is generally not practicable leading to high false-negative results in neural tracking. In this study, we evaluated the usefulness of the neurite orientation dispersion and density imaging (NODDI) derived tractography for investigating structural heterogeneity of the brain in patients with brain tumor. A total of 24 patients with brain tumors, characterized by peritumoral edema, and 10 healthy counterparts were recruited from 2014 to 2021. All participants underwent magnetic resonance imaging. Moreover, we used the images obtained from the healthy participants for calibrating the orientation dispersion threshold for NODDI-derived corticospinal tract (CST) reconstruction. Compared to DTI, NODDI-derived tractography has a great potential to improve the reconstruction of fiber tracking through regions of vasogenic edema. The regions with edematous CST in NODDI-derived tractography demonstrated a significant decrease in the intracellular volume fraction (VF_ic_, *p* < 0.000) and an increase in the isotropic volume fraction (VF_iso_, *p* < 0.014). Notably, the percentage of the involved volume of the concealed CST and lesion-to-tract distance could reflect the motor function of the patients. After the tumor resection, four patients with 1–5 years follow-up were showed subsidence of the vasogenic edema and normal CST on DTI tractography. NODDI-derived tractography revealed tracts within the edematous area and could assist neurosurgeons to locate the neural tracts that are otherwise not visualized by conventional DTI tractography.

## Introduction

The cerebral neoplasm may increase the intracranial pressure and cause neural tract deviation, infiltration, or destruction in the site of tumor growth and peritumoral areas (Assaf and Pasternak, 2008). Various magnetic resonance imaging (MRI) techniques are used to access and localize brain tumors to obtain a thorough resection and minimize functional loss. Among these techniques, diffusion tensor imaging (DTI) has been widely used to demonstrate the structural contents and peritumoral neural tracts. DTI is a non-invasive method that can probe the molecular diffusivity of water within the white matter to reflect the intravoxel architecture by measuring the water self-diffusion tensor (Basser et al., 1994; Basser, 1995). Herein, linking the anisotropic orientation determined by the principal eigenvector of the tensor has been widely applied to map the neuronal tracts (Mori et al., 1999; Mori and Van Zijl, 2002), which has also provided crucial information to neurosurgeons for neurosurgical planning and navigation (Berman et al., 2004; Bello et al., 2008). However, as a simplified model, DTI-derived tractography faces a substantial challenge in resolving the fiber tract within a voxel that comprises heterogeneous compartments including infiltrating cells and edematous tissues (Lecoeur et al., 2014; Chen et al., 2016; Gong et al., 2018; Ye et al., 2020).

Most aggressive brain tumors, such as malignant gliomas and metastatic tumors, are usually associated with peritumoral vasogenic edema (Lu et al., 2003). The latter generally infiltrates brain tissues with a lot of body fluid and alters the measures of diffusion characteristics, increasing the uncertainty of presurgical evaluation and leading to an enormous impact on patient management and surgical planning (Assaf and Pasternak, 2008; Lecoeur et al., 2014). Thus, it is crucial to identify the constituents of the infiltrated tissue and perform tractography within the area. However, the use of single ellipsoid DTI model makes this process challenging (Zhang et al., 2013). According to diffusion physics, diffusion-weighted images (DWI) with high *b*-value encoding can quantify the structural architecture and resolve structural heterogeneity (Callaghan et al., 1991; Lecoeur et al., 2014; Ye et al., 2020). Bi-exponential and higher-order models ascribe magnetic resonance (MR) signal attenuation into two processes, namely, restricted and hindered water diffusion in the intracellular and extracellular spaces, respectively, and provide a unique opportunity to reveal neural tracts within the hydro-environment compared with the conventional method. Previous studies have mentioned that multi-compartment diffusion models, such as the free water model (Lecoeur et al., 2013) and two-tensor unscented Kalman filter (UKF) (Chen et al., 2015, 2016) improve the tracking of the neuronal fiber in peritumoral vasogenic edema. This suggests that fiber tracking in the edema would benefit from the prior heterogeneity. Knowing the microstructure change in the peritumoral region may further help identify whether the fiber still existed, thus suggesting the need for an advanced biophysical diffusion model to explore the edematous fiber tracts (Liao et al., 2017).

Neurite orientation dispersion and density imaging (NODDI) is a clinically feasible three-compartment model that utilizes two *b*-value encodings (Zhang et al., 2012). The aforementioned model is capable of estimating three types of water diffusion behavior with different volume fractions, i.e., restricted (intracellular volume fraction), hindered (extracellular volume fraction), and free diffusion (isotropic volume fraction). These indices provide special measures that represent the axonal density and the dispersion of fiber orientations. NODDI is reportedly valuable for evaluating various neurological diseases, such as white matter microstructural changes in Alzheimer’s disease (Fu et al., 2020) and Parkinson’s disease (Mitchell et al., 2019). Moreover, it provides distinctive markers for multiple sclerosis (Crombe et al., 2018) and stroke (Adluru et al., 2014; Wang et al., 2019). Numerous studies have suggested that NODDI indices could provide unique contrast in brain tumors (Wen et al., 2015), which will help to differentiate between glioblastoma and solitary brain metastasis and reveal the differences between tumor infiltration and vasogenic edema (Kadota et al., 2020). Moreover, the NODDI indices are capable of characterizing white matter fibers involved in tumoral and edematous brain areas (Masjoodi et al., 2018), which implies that this multi-compartment model may potentially demonstrate neural tractography in peritumoral edema.

Although the peritumoral neural tracts involved in motor functions are crucial to neurosurgery, they are generally concealed by vasogenic edema and usually unseen with traditional diffusion modeling. Therefore, in this study, we aimed to use the NODDI model to investigate the structural heterogeneity in patients with vasogenic edema. First, we reconstructed the edematous corticospinal tract (CST) that were unseen with traditional diffusion modeling. Second, we provided evidence that the concealed tracts could reflect motor function, such as muscle strength, which would benefit surgical planning. Third, white matter integrity profiles were evaluated by the multi-compartment characteristics for better understanding the microstructure heterogeneity of the region of edema. Finally, we examined the disclosed tracts that could be detected following the release of the vasogenic edema.

## Materials and Methods

### Participants

We recruited a total of 24 patients with brain tumor and 10 healthy participants from 2014 to 2021. The enrolled patients were diagnosed with cerebral neoplasm and a large volume of perilesional vasogenic edema in the motor area, primary somatosensory cortex, or premotor area that affected the CST. The vasogenic edema was determined by peritumoral hyperintensity on T2-weighted fluid-attenuated inversion recovery (T2-FLAIR) images and no evidence of restricted water diffusion on diffusion-weighted imaging (DWI). Each patient underwent muscle strength measurement by using the Medical Research Council (MRC) scale of muscle strength. Motor function decline was defined as abnormal if the MRC grading was 4 or less. This study was approved by the local institutional review board, and all participants provided informed consent for the surgery.

### MRI Acquisition and Imaging Preprocessing

#### Data Set I

The first dataset enrolled six patients and five healthy participants at the Tri-Service General Hospital, Taipei, Taiwan. MRI data were acquired on a 3-T GE machine (Discovery MR 750, GE Healthcare, Milwaukee, United States) with an eight-channel head coil. The clinical routine MR scanning protocols included a high-resolution 3-D Fast Spoiled Gradient Echo T1-weighted images (T1-weighted images (T1WI); field of view (FOV) = 224 × 224 × 146 mm^3^; voxel size = 1 × 1 × 1 mm^3^; and TE/TR/TI = 4.2/10.2/450 ms), 2-D axial view fast recovery fast spin-echo T2-weighted images (T2-weighted images (T2WI); FOV = 250 × 250 × 150 mm^3^; voxel size = 0.49 × 0.49 × 6 mm^3^; and TE/TR = 100.7/5,619 ms), 2-D axial view FSE T2-FLAIR (FOV = 250 × 250 × 150 mm^3^; voxel size = 0.49 × 0.49 × 6 mm^3^; and TE/TR/TI = 118.3/12,000/2,200 ms), and 2-D axial view T1WI following the injection of gadolinium-based contrast agent (gadolinium-enhanced T1WI (Gd-T1WI); FOV = 250 × 250 × 150 mm^3^; voxel size = 0.49 × 0.49 × 6 mm^3^; and TE/TR/TI = 31.1/3,465.2/928.8 ms). Two-shelled DWI were acquired using a single-shot spin-echo planer imaging sequence (FOV = 240 × 240 × 112 mm^3^; voxel size = 2.5 × 2.5 × 2.5 mm^3^; TE/TR = 88/6,000 ms; four null images; 30 directions of b = 1,000 s/mm^2^; and 60 directions of b = 2,000 s/mm^2^).

#### Data Set II

The second dataset included 18 patients and five healthy participants at The First Hospital of Jilin University, Jilin, China. MRI data were acquired on a 3-T Siemens machine (Magnetom Verio, Siemens, Erlangen, Germany) with a 16-channel head coil. The clinical MR scanning protocol included a 2-D axial view T1WI (FOV = 230 × 230 × 117 mm^3^; voxel size = 0.36 × 0.36 × 6.5 mm^3^; and TE/TR/TI = 9/1,500/791.8 ms), 2-D axial view T2WI (FOV = 230 × 230 × 117 mm^3^; voxel size = 0.6 × 0.6 × 6.5 mm^3^; and TE/TR = 95/6,000 ms), 2-D axial T2-FLAIR (FOV = 230 × 230 × 117 mm^3^; voxel size = 0.72 × 0.72 × 6.5 mm^3^; and TE/TR/TI = 81/7,500/2,297.9 ms), and 3-D magnetization-prepared rapid gradient-echo Gd-T1WI (FOV = 250 × 250 × 160 mm^3^; voxel size = 1 × 0.98 × 0.98 mm^3^; and TE/TR/TI = 3.5/2,300/1,070 ms). Two-shelled DWI were acquired using a single-shot spin-echo planer imaging sequence (FOV = 250 × 250 × 160 mm^3^; voxel size = 2.5 × 2.5 × 2.5 mm^3^; and TE/TR = 95/16,700 ms; four null images; 30 directions of b = 1,000 s/mm^2^; and 60 directions of b = 2,000 s/mm^2^). We also acquired three null diffusion images with opposite polarity (phase encoding form posterior to anterior).

#### Imaging Preprocessing

**FIGURE 1:**
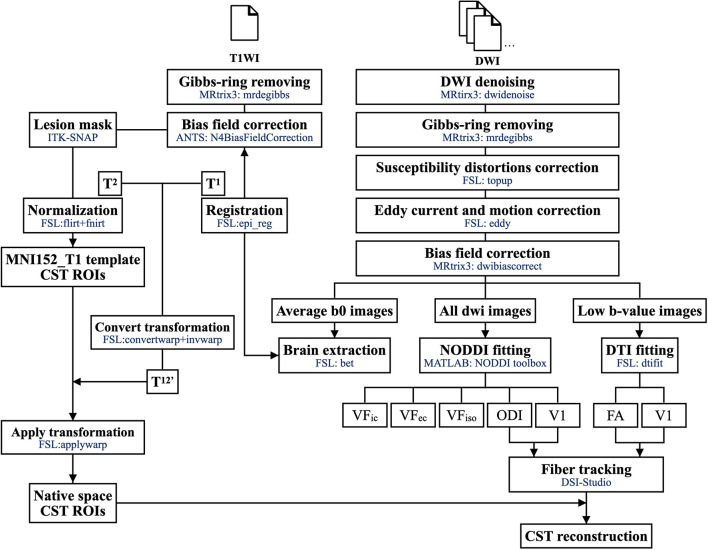
The imaging processing workflow.

All DWI data were processed by the in-house developed DWI preprocess pipeline (OGIO) that includes algorithms and functions from MRtrix3^1^ FMRIB Software Library (FSL)^2^ and ANTs^3^. The overall imaging preprocessing workflow is summarized in Figure 1.

First, we used dwidenoise (Veraart et al., 2016a,b; Cordero-Grande et al., 2019) and mrdegibbs (Kellner et al., 2016) functions in MRtrix3 (Tournier et al., 2019) for imaging denoising and removing the Gibbs ringing artifact from the DWI. To correct the susceptibility-included off-resonance field, image distortion induced by the fast-switching gradient, slight head motion, and FSL functions (Jenkinson et al., 2012) TOPUP and EDDY (Graham et al., 2017) were performed on DWIs acquired with two opposite polarities. Finally, dwibiascorrect (Tustison et al., 2010) was utilized to correct performing B1 field inhomogeneity in the DWI volume series. The Brain Extraction Tool function (Smith, 2002) from FSL was used to create a brain mask from the average null DWIs. For T1WI, we used the mrdegibbs (Kellner et al., 2016) function for removing the Gibbs ringing artifact and N4BiasFieldCorrection (Tustison et al., 2010) from ANTS for B1 field inhomogeneity correction.

### Model Fitting

**FIGURE 2:**
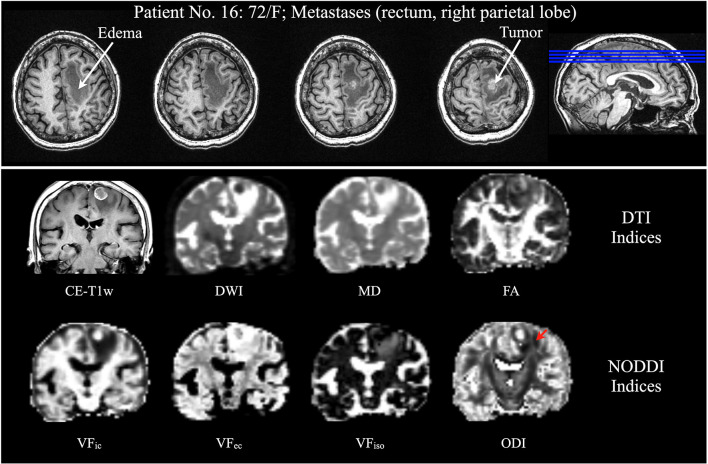
Patient No. 16, a 72-year-old woman with rectum metastasis in the right parietal lobe. (Top) Axial view of CE-T1WI displays an enhancing tumor in the right superior frontal lobe, with significant vasogenic edema. (Bottom) Coronal view of CE-T1WI, DWI, DTI indices (MD, and FA), and NODDI indices (VF_*ic*_, VF_*ec*_, and VF_*iso*_) could not reveal the CST except the ODI from NODDI (red arrow). CE-T1WI, contrast enhanced T1-weighted images; DWI, diffusion-weighted imaging; DTI, diffusion tensor imaging; MD, mean diffusivity; FA, fractional anisotropy; NODDI, neurite orientation dispersion and density imaging; VF_*ic*_, intracellular volume fraction; VF_*ec*_, extracellular volume fraction; and VF_*iso*_, isotropic volume fraction; CST, corticospinal tract; and ODI, orientation dispersion index.

All images were fitted with the NODDI model using the NODDI MATLAB toolbox^4^. The low-*b*-value images (<1,500 s/mm^2^) were used in DTI model fitting (with dtifit in FSL). We subsequently obtained the voxel-wise maps. We evaluated the first eigenvector for the principal directions on DTI tractography and used fractional anisotropy (FA) as the fiber tractography termination criteria. For NODDI-derived indices, we calculated the orientation dispersion index (ODI), fiber orientation (the principal directions from NODDI model), and the three absolute volume fractions of NODDI (intracellular volume fraction, VF_*ic*_; extracellular volume fraction, VF_*ec*_; isotropic volume fraction, VF_*iso*_), where VF_*ic*_ + VF_*ec*_ + VF_*iso*_ = 1 (Figure 2).

### ROI Selection

**FIGURE 3:**
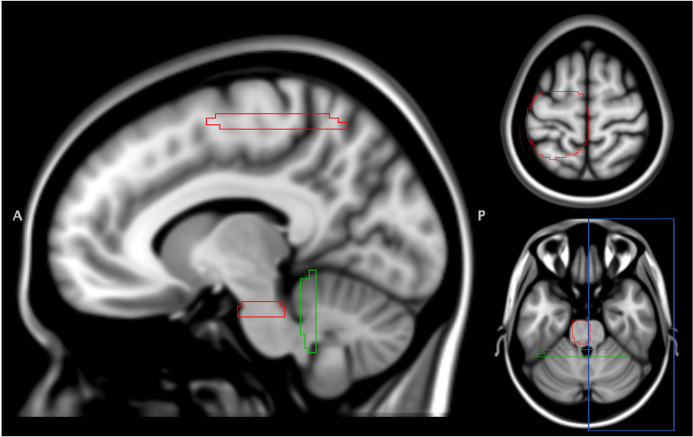
Regions of interest for CST extraction. A total of 4 ROIs were defined in the MNI152 T1 space. Two ROIs (sensorimotor area and brainstem, red) were used for CST extraction. Another two ROIs for excluding streamlines into cerebellum (green) and cross-hemisphere connection (blue). ROI, region of interest; CST, corticospinal tract.

For CST extraction, four regions of interest (ROIs) were manually drawn in the Montreal Neurological Institute (MNI) space according to previous study (Cooper et al., 2015). The first and second ROIs (axial view of sensorimotor area and brainstem) were used for CST extraction. The third and fourth ROIs were the exclusion ROIs for excluding streamlines into the cerebellum and cross-hemisphere connection (Figure 3). Following an inspection of T1WI, T2WI, T2-FLAIR, DWI, and Gd-T1WI, the ROIs of lesion and edema for each patient were manually drawn by using ITK-SNAP^5^ (Yushkevich et al., 2006). All ROIs were manually drawn by a neuroradiologist (H-W Kao) with 15 years of experience.

### Imaging Registration

To transform the ROIs from the MNI standard space to the individual space, T1WI was first co-registered with average null diffusion images using boundary-based registration to generate the transformation matrix from the DWI space to the T1WI space. Second, T1WI was then spatially normalized to the MNI152 T1 template in the standard space via linear affine transformation (FLIRT) and non-linear registration (FNIRT) (Zhang et al., 2001; Smith et al., 2004; Patenaude et al., 2011). The lesion mask was used to exclude the calculation in the nonlinear registration. Combining the two transformation matrices (DWI to T1WI and T1WI to MNI), we applied the inverse transformation matrix to obtain the ROIs in each individual’s native DWI space with the nearest-neighbor interpolation. All the spatial registration and normalization procedures were implemented in FSL.

### Fiber Tractography and ODI Threshold Calibration

CST was reconstructed from the directions identified by both DTI and NODDI. The NODDI-derived CSTs were reconstructed by modifying the FIB file of DSI-studio with NODDI’s ODI and fiber orientation. All fiber tractography was done using the deterministic fiber algorithm (Yeh et al., 2013) implanted in the DSI-Studio^6^. Four ROIs (two for inclusion and two for exclusion, shown in Figure 3) were utilized for CST extraction. The step size was 0.5 mm; streamlines with lengths shorter than 30 mm or longer than 300 mm were discarded. A total of 3,000 streamlines were finally reconstructed.

To calibrate the ODI threshold, 10 healthy participants (five from each site) were included. We first reconstructed the DTI-derived CST with standard tracking termination criteria [fractional anisotropy (FA) ≤ 0.20; turning angle ≥ 60°] (Mori et al., 1999). Next, the NODDI-derived CSTs were reconstructed with ODI thresholds varying from 0.2 to 1. Moreover, the streamline similarity was evaluated using the dice similarity index (DSI) between DTI tractography and each ODI threshold CST. The DSI was defined as follows: $D\,S\,I=\frac{2\,\left(D\,T\cap N\,O\,D\,D\,I\right)}{D\,T\,I+N\,O\,D\,D\,I}$. The optimal ODI threshold was determined with the maximized overlap.

In this study, assessment of the differences between DTI and NODDI-derived tractography was achieved by comparing the DTI-derived CST with the standard FA threshold (FA ≤ 0.20), DTI-derived CST with low FA (FA ≤ 0.10), and NODDI-derived CST with optimal ODI threshold on 24 patients.

### Statistical Analyses

**FIGURE 4:**
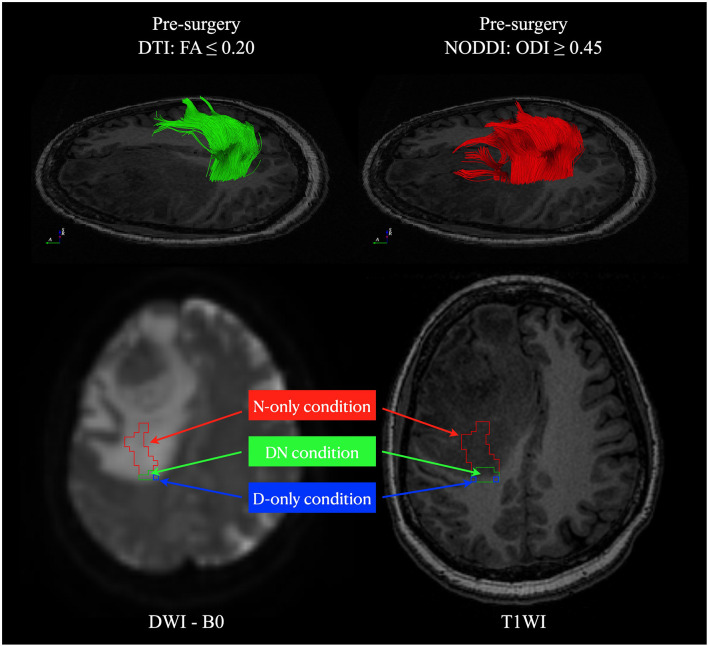
The fiber tracts volume condition definition. Three conditions of the fiber tracts located in areas overlapping with peritumoral edema were classified in each patient: (1) DN condition: the volume of both DTI and NODDI-derived fiber tracts in the peritumoral edema (green); (2) D-only condition: the volume of only DTI fiber tracts in the peritumoral edema (blue); (3) N-only condition: the volume of only NODDI-derived fiber tracts in the peritumoral edema (red). DTI, diffusion tensor imaging; NODDI, neurite orientation dispersion and density imaging.

To evaluate the microstructure changes in the edema region, we applied the edema mask for extracting the voxels with streamline pass through. The following three conditions of edematous fiber tracts were classified in each patient: (1) DN condition, the volume of both DTI and NODDI-derived fiber tracts in the peritumoral edema; (2) D-only condition, the volume of only DTI fiber tracts in the peritumoral edema; and (3) N-only condition, the volume of only NODDI-derived fiber tracts in the peritumoral edema (Figure 4). We calculated the percentage of involved volume (PIV) in the three conditions of CST to assess the patient’s motor function. PIV was defined as follows: $P\,I\,V=\frac{v\,o\,l\,u\,m\,e\,o\,f\,c\,o\,n\,d\,i\,t\,i\,o\,n}{v\,o\,l\,u\,m\,e\,o\,f\,C\,S\,T}$. The volume of CST was defined as the total volume of CST in DTI and NODDI-derived reconstruction. Moreover, we measured the lesion-to-tract distance (LTD) from the lesion boundaries to the nearest CST in a three-dimensional view with the in-house-developed viewer (GoViewer). To integrate the evaluation index with LTD, the volume and PIV of each condition were divided by LTD as weighted.

We determined the diagnostic performance for differentiating the motor function using the receiver operating characteristic (ROC) curves. We calculated the sensitivity, specificity, and area under the ROC curve (AUC) to determine the diagnostic performance of each index. Moreover, Youden’s *J* statistic was used for determining the optimal cutoff threshold for the ROC curve. The Youden index is defined as *J*_*max*_=*max*_*t*_{*sensitivity*(*t*) + *specificity*(*t*)−1}, where *t* denotes the classification threshold for which *J* is maximal from the ROC curve. Furthermore, we extracted the mean NODDI-derived volume fractions (VF_*ic*_, VF_*ec*_, and VF_*iso*_) in DN and N-only conditions. The Wilcoxon signed rank test was conducted to evaluate the differences in the microenvironment between the two conditions. All statistical analyses were analyzed using statistical software (SPSS, version 25; SPSS, Chicago, Ill). *p*-values < 0.05 were considered statistically significant.

### Follow-Up Comparison

A total of four patients were recruited after imaging showing subsidence of the vasogenic edema: two patients with 1 year (from data set II) and another two with 5 years (from data set I) after the tumor resection. MRI data acquisition and imaging processing were the same as the initial. The DTI tractography of CST reconstruction was performed with the standard FA criteria (FA ≤ 0.20; turning angle ≥60°). To quantify the restoration of the CST in the follow-up compared to initial DTI- and NODDI-derived tractography, we compared the fiber tractography between pre- and post-surgery. We registered the pre-surgery and follow-up data by using linear registration (FLIRT). DSI was used for the streamline similarity comparison.

## Results

The study included a total of 24 patients (9 men and 15 women). The mean age was 52 years, ranging from 32 to 72 years. Seven patients were diagnosed with meningiomas (four and three with grades I and II, respectively). Six patients had gliomas (one with grade I, one with grade II, and two each with grades III and IV). Six patients had metastases (two lung cancers, two rectal cancers, and two cancers of unknown origin). The other five patients had lymphoma, primitive neuroectodermal tumor, Ewing’s sarcoma, abscess, and gliosis. Among our patients, six patients experienced motor function decline. Table 1 summarizes the clinical information, imaging features, LTD, and pathological diagnoses of all patients.

**TABLE 1:** A summary of the clinical information, imaging features, and pathology of 24 patients.

					**SI of solid portion**		**CE of solid portion**			**Lesion to tract distance**	**Motor function decline**
**Case**	**Age/sex**	**Location**	**Edema**	**Edema volume**	**Gd–T1WI**	**T2WI**		**Pathology**	**WHO grading**		
1	65/F	Left frontal	S	18,658 mm^3^	Hyper	Hyper	Hete	Meningioma	Grade I	37.2 mm	No
2	42/M	Right parietal	Mo	8,282 mm^3^	Hyper	Iso	Homo	Meningioma	Grade I	12.9 mm	Yes
3	49/F	Left frontal	Mo	10,648 mm^3^	Hyper	Iso	Homo	Meningioma	Grade I	17.4 mm	No
4	38/M	Right parietal	S	24,115 mm^3^	Hyper	Hyper	Homo	Meningioma	Grade I	61.3 mm	No
5	36/M	Left frontal	S	29,935 mm^3^	Hyper	Iso	Hete	Meningioma	Grade II	40.6 mm	No
6	57/F	Left frontal	S	27,435 mm^3^	Hyper	Hyper	Homo	Meningioma	Grade II	23.5 mm	No
7	55/F	Right frontal	Mo	12,283 mm^3^	Hyper	Iso	Homo	Meningioma	Grade II	16.4 mm	No
8	32/F	Right frontal	S	26,603 mm^3^	Hypo	Iso	Hete	Glioma	Grade I	21.6 mm	No
9	43/M	Right parietal	Mo	1,870 mm^3^	Hypo	Hyper	Homo	Glioma	Grade II	11.3 mm	No
10	56/M	Right frontal	S	17,733 mm^3^	Hypo	Hyper	Homo	Glioma	Grade III	5.0 mm	No
11	55/F	Right parietal	Mo	9,557 mm^3^	Hypo	Hyper	Homo	Glioma	Grade III–IV	10.0 mm	No
12	37/F	Right frontal	S	33,488 mm^3^	Hyper	Hyper	Hete	Glioblastoma	Grade IV	19.4 mm	No
13	72/M	Left temporal	Mo	12,180 mm^3^	Hyper	Hyper	Hete	Glioblastoma	Grade IV	3.8 mm	No
14	51/F	Right frontal	S	24,288 mm^3^	Hyper	Hypo	Hete	Metastatic (lung)	Grade IV	34.7 mm	No
15	51/F	Left parietal	S	15,738 mm^3^	Hyper	Hyper	Hete	Metastatic (lung)	Grade IV	4.8 mm	Yes
16	72/F	Right parietal	Mo	6,045 mm^3^	Hyper	Hypo	Homo	Metastatic (rectum)	Grade IV	4.6 mm	Yes
17	69/F	Right frontal	S	30,948 mm^3^	Hyper	Hypo	Hete	Metastatic (adenocarcinoma)	Grade IV	36.5 mm	No
18	59/F	Right frontal & temporal	Mo	5,535 mm^3^	Hyper	Hypo	Hete	Metastatic (unknown)	Grade IV	15.2 mm	No
19	64/M	Left parietal	S	1,7875 mm^3^	Hyper	Hypo	Hete	Metastatic (unknown)	Grade IV	16.4 mm	Yes
20	38/M	Right parietal	Mo	7,207 mm^3^	Hypo	Hyper	Homo	Primitive neuroectodermal tumor	Grade IV	8.7 mm	Yes
21	53/F	Left frontal	S	21,523 mm^3^	Hyper	Hypo	Homo	Malignant B-cell lymphoma	–	40.2 mm	No
22	55/F	Left parietal	Mo	8,680 mm^3^	Hyper	Hypo	Hete	Ewing’s sarcoma	–	7.7 mm	No
23	36/F	Left frontal	S	15,620 mm^3^	Hyper	Hyper	Hete	Gliosis	–	2.3 mm	Yes
24	65/M	Left parietal	S	19,889 mm^3^	Hyper	Hypo	Hete	Abscess	–	12.9 mm	Yes

*CE, contrast enhancement; Hete, heterogeneous; Homo, homogeneous; Hyper, hyperintensity; Hypo, hypointensity; Iso, isointensity; Mo, moderate; S, severe; SI, signal intensity.*

### ODI Threshold Calibration

**FIGURE 5:**
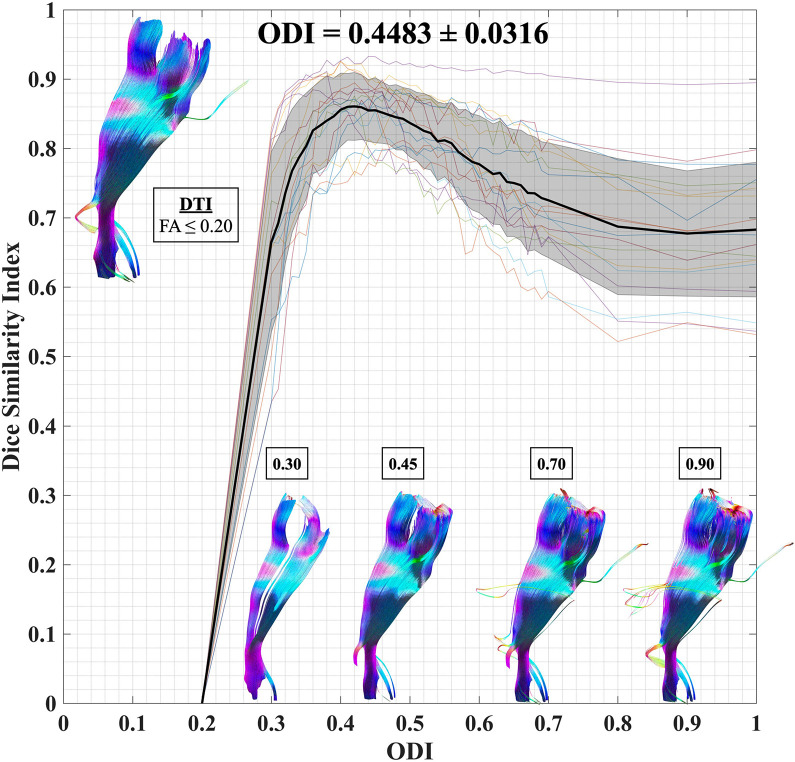
ODI threshold calibration for CST. We have studied the dice similarity index at each considered ODI threshold compared to the standard DTI termination criteria (FA ≤ 0.2; turning angle ≥ 60°). An optimal ODI ≥ 0.45 has been set for CST reconstruction. ODI, orientation dispersion index; CST, corticospinal tract; and DTI, diffusion tensor imaging.

The mean and maximum DSI values of CST reconstruction in the healthy participants were 0.8077 and 0.9164, respectively, with ODI ≥ 0.4482 ± 0.0316. We determined an optimal ODI threshold of 0.45 (Figure 5).

### Comparison Between DTI and NODDI

**FIGURE 6:**
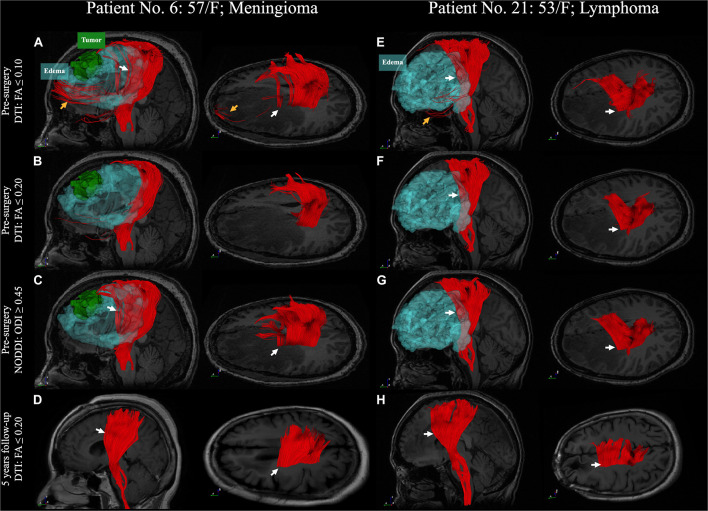
A comparison between DTI and NODDI-derived CST reconstruction with 5 years follow-up. **(A–D)** Patient No. 6, a 57-year-old woman with a meningioma (WHO grade II) in the left frontal lobe. **(E–H)** Patient No. 21, a 53-year-old woman with malignant B-cell lymphoma. DTI tractography with standard termination criteria (FA ≤ 0.2) has failed to reconstruct the CST in the region with peritumoral edema in the first case. **(B)** However, the CST has been successfully reconstructed in the second case (**F**, white arrow). DTI tractography with low FA criteria (FA ≤ 0.1) displays better performance in the region with peritumoral edema (white arrow). Nonetheless, it is accompanied by noisy streamlines (**A,E**, yellow arrow). NODDI-derived tractography with ODI reveals better performance in the region with edema and less noisy streamlines (**C,G**, white arrow). The DTI tractography with standard termination criteria reveals a CST similar to the pre-surgery NODDI-derived CST after the vasogenic edema has subsided in 5 years **(D,H)**. DTI, diffusion tensor imaging; FA, fractional anisotropy; NODDI, neurite orientation dispersion and density imaging; CST, corticospinal tract; WHO, World Health Organization and ODI, orientation dispersion index.

**FIGURE 7:**
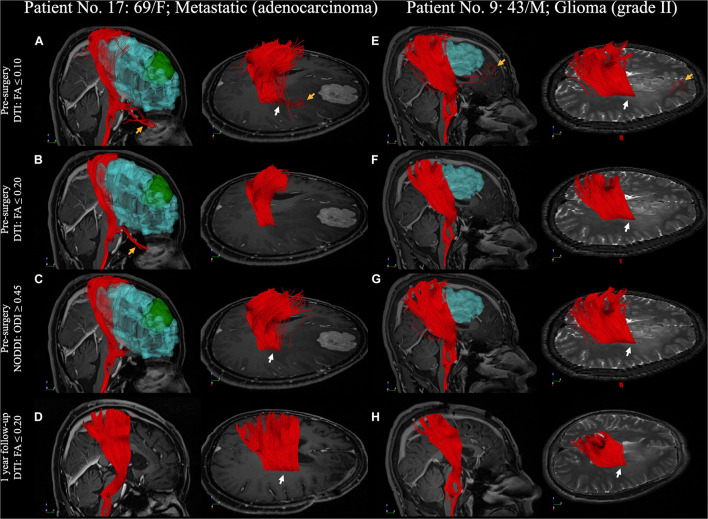
A comparison between DTI and NODDI-derived CST reconstruction with 1-year follow-up. **(A–D)** Patient No. 17, a 69-year-old woman with an adenocarcinoma metastatic in the right frontal lobe. **(E–H)** Patient No. 9, a 43-year-old man with WHO grade II glioma in right parietal lobe. CST reconstruction in DTI and NODDI-derived tractography in pre-surgery and 1 year after tumor resection. White arrow indicated the fiber tracts in the peritumoral edema. DTI, diffusion tensor imaging; FA, fractional anisotropy; NODDI, neurite orientation dispersion and density imaging; CST, corticospinal tract; WHO, World Health Organization and ODI, orientation dispersion index.

We compared the differences between DTI and NODDI, predominantly found in regions with vasogenic edema. DTI-derived tractography with standard FA displayed non-stabilized performance in the regions of vasogenic edema, comprising only sparse tracts. DTI tractography with low FA revealed better performance but was accompanied by several noisy streamlines. NODDI-derived tractography could reveal the CST in the regions of vasogenic edema, with fewer noisy streamlines (Figures 6, 7). Two patients (patient nos. 2 and 15) failed to reconstruct any of the CST in the regions with vasogenic edema while using DTI tractography (Supplementary Figure 1).

### ROC Diagnostic Performance Analysis

**FIGURE 8:**
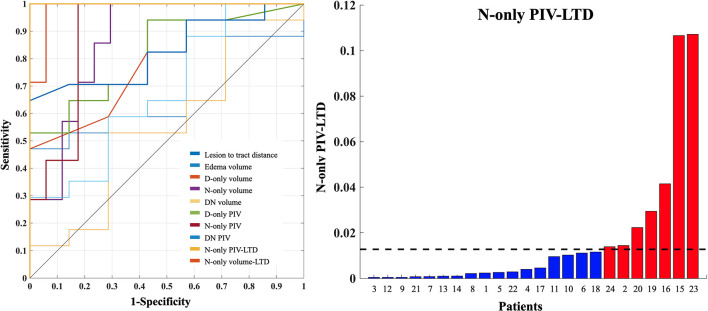
The PIV-LTD of N-only condition and ROC diagnostic performance of motor functions. The PIV of N-only condition weighted by LTD had the best diagnostic performance for determining the presence of motor function decline (left, gold line). An optimal threshold of PIV-LTD of N-only condition >0.0128 has yielded a sensitivity, specificity, and AUC of 100%, 100%, and 1 for differentiating patients with motor function decline (right). The red and blue color bars indicate patients with and without motor function decline. The black dash line indicates the optimal threshold, determined by using the Youden index. PIV, percentage of involved volume; LTD, lesion-to-tract distance; ROC, receiver operating characteristic; AUC, area under the receiver operating characteristic curve.

In ROC diagnostic performance analysis, we found that LTD, D-only volume, D-only PIV, N-only volume, N-only volume-LTD (N-only volume with weighted LTD), N-only PIV, and N-only PIV-LTDs (N-only PIV with weighted LTD) had significant differences between patients with and without surgery-related paresis. The PIV of the N-only condition weighted by LTD had the best diagnostic performance for determining the presence of motor function decline. The threshold value of >0.0128 yielded a sensitivity, specificity, and AUC of 100, 100%, and 1, respectively, for patients with motor function decline (Figure 8). The threshold value and diagnostic performance of each index in motor function decline are summarized in Table 2. The statistical details of group comparison between patients with and without motor function decline can be found in Supplementary Table 2.

**TABLE 2:** The threshold value and diagnostic performance in motor function decline.

	**Threshold value**	**Sensitivity**	**Specificity**	**AUC**	**Standard error**	**Significant**	**95% of confidence interval**
Edema volume	20,260 mm^3^	0.471	1	0.689	0.109	0.153	0.475–0.903
D-only volume	3.75 mm^3^	0.941	0.571	0.798	0.096	0.024	0.609–0.987
N-only volume	665 mm^3^	1	0.06	0.866	0.073	0.006	0.722–1
DN volume	2,281 mm^3^	0.529	0.714	0.555	0.136	0.680	0.289–0.820
D-only PIV	0.035	0.529	1	0.815	0.090	0.017	0.638–0.992
N-only PIV	0.1742	1	0.824	0.891	0.067	0.003	0.759–1
DN PIV	0.0125	0.882	0.429	0.681	0.122	0.172	0.441–0.921
N-only volume-LTD	54.75	1	0.941	0.983	0.021	0.000	0.941–1
N-only PIV-LTD	0.0128	1	1	1	0	0.000	1–1
LTD	16.9 mm	0.647	1	0.828	0.084	0.013	0.664–0.992

*PIV, percentage of involved volume; LTD, lesion-to-tract distance; AUC, area under the receiver operating characteristic curve.*

### NODDI Fraction Comparison

**FIGURE 9:**
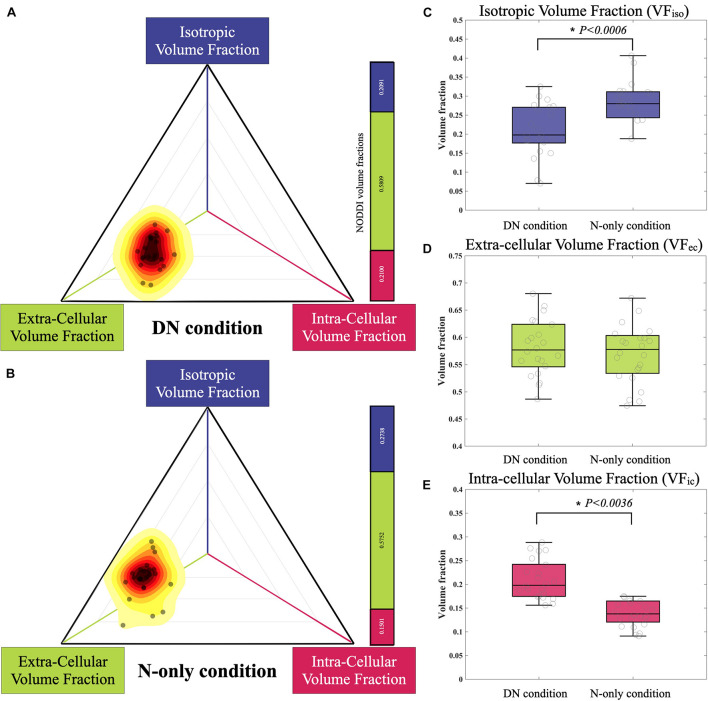
The distribution of NODDI-derived volume fraction in the DN condition and N-only condition are plotted with a 2-D barycentric coordinate system. **(A)** The N-only condition and **(B)** distribution reveal a left upper shift in the coordinate system, compared to the DN condition. There is no significant difference in the VF_*ec*_ (*p* = 0.4751, **D**). However, there are significant differences in the VF_*ic*_ (*p* < 0.0036, **E**) and VF_*iso*_ (*p* = 0.0006, **C**). NODDI, neurite orientation dispersion and density imaging.

In a 2-D barycentric coordinate system, the distribution of the N-only condition (mean VF_*ic*_ = 0.1501; mean VF_*ec*_ = 0.5752; and mean VF_*iso*_ = 0.2738) was presented in the left upper shift relative to that in the DN condition (mean VF_*ic*_ = 0.2100; mean VF_*ec*_ = 0.5809; and mean VF_*iso*_ = 0.2091). The statistical comparison of NODDI indices between the N-only condition and DN condition showed no significant difference in VF_*ec*_ (*p* = 0.4751). Moreover, we observed a significant increase and decrease of VF_*iso*_ (*p* < 0.0006) and VF_*ic*_ (*p* < 0.0036) in the N-only condition when comparing it to the DN condition (Figure 9).

### Tract Verifications After Releasing the Vasogenic Edema

**FIGURE 10:**
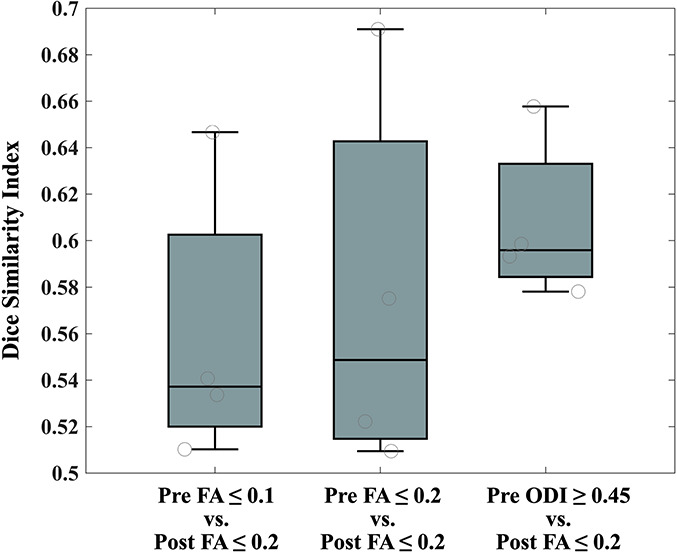
The similarity comparison of CST reconstruction between pre- and post-surgery. CST, corticospinal tract.

After the tumor resection, two patients with 5-year follow-up (Figure 6) and two with 1-year follow-up (Figure 7) showed reduced vasogenic edema and normal CST on DTI tractography with standard termination criteria (FA ≤ 0.20). The similarity comparison showed that NODDI-derived tractography performed the highest similarity and lowest standard deviation (0.607 ± 0.035) with post-surgery fiber tractography (Figure 10).

## Discussion

Specific functional tracts like CST that are concealed by peritumoral edema are meaningful to neurosurgeons. However, they have not yet been systematically disclosed. We applied an advanced NODDI model to different diffusion contents to highlight the neural anisotropy and tractography. Compared to conventional DTI tractography, NODDI-derived fiber tractography provided better performance in heterogeneous areas. Notably, the involved volume of the concealed tracts and LTD directly reflected their functional performance, which, in turn, would facilitate pre-surgery planning and post-surgery recovery assessment. After the vasogenic edema subsided, we verified the neural tracts in four patients during the 1–5-year follow-up.

DTI tractography effectively maps neural tracts within the human brain. However, it has been proven unreliable in revealing the fiber tracts in the peritumoral edema region (Zhang et al., 2013; Ye et al., 2020). Nonetheless, specific functional fiber tracts that are located in areas overlapping with peritumoral edema are crucial to the functional protection of the patients. Previous studies have mentioned that the single-tensor model has a high probability of failure in revealing the fiber tracts when the image voxel gets affected by the vasogenic edema (partial volume effect) (Lecoeur et al., 2013; Zhang et al., 2013; Liao et al., 2017). This necessitates the deconstruction of DWI signals. Lecoeur et al. (2013) proposed removing the free water compartment for tensor correction and achieved significantly improved tractography in the presence of edema. This concept was extended to further studies, such as free water modeling (Gong et al., 2018), two-tensor unscented UKF (Chen et al., 2015, 2016; Liao et al., 2017), and DBSI (Ye et al., 2020). Following the exclusion of the free water compartment, the above-mentioned models demonstrated the possibility of tracing a larger volume of fiber bundles. Derived from the NODDI model, the ODI summarized the angular variation of neurites by excluding the isotropic diffusivity. Therefore, the ODI showed a more apparent trend in fiber direction in the area with vasogenic edema when compared to the DTI-derived FA map (Figure 2). It also displayed higher sensitivity and specificity of CST reconstruction in the NODDI-derived tractography than did DTI tractography (Figure 6). We specified on CST reconstruction to systematically compare the results and validate them through follow-up data. Previous studies have suggested that the vasogenic edema may be resolved in 4 days after total removal and that it disappeared entirely by 2–3 weeks post-operation (Stevens et al., 1983; Shirotani et al., 1994). Despite a follow-up in only four patients, the tractography results and the similarity comparison suggested that free water fraction exclusion could improve fiber tracking in regions with vasogenic edema.

Conventionally, the FA threshold, one of the stopping criteria for DTI tractography, was chosen rather arbitrarily, ranging from 0.15 to 0.30 for general tracking purposes (Stadlbauer et al., 2007; Bello et al., 2008). Stadlbauer et al. (2007) conducted a study on brain tumor infiltration and associated vasogenic edema. They demonstrated that an increase in the FA threshold stepped up the distance between the reconstructed fiber bundles and the tumors and decreased the number of detectable fiber bundles. Accordingly, the FA threshold was usually selected at 0.15 or 0.10 to display the peritumoral neural tracts (Bello et al., 2008). Despite the high tracking sensitivity of low FA, low specificity increases the probability of noisy streamline reconstruction. This, in turn, concurrently increases the uncertainty of neurosurgery. Therefore, surgeons should be more careful while applying the results mentioned above in surgical planning and navigation.

Following the exposure of the fiber tracts located in areas overlapping with peritumoral edema, we calculated the CST volume and LTD associated with motor functions. Numerous studies have suggested that the CST volume may reflect clinical outcomes, such as stroke recovery (Auriat et al., 2015) and amyotrophic lateral sclerosis (Wang et al., 2006). Sollmann et al. (2019) and Yu et al. (2020) suggested that the LTD was related to surgery-related transient impairment and may well be regarded as a presumptive predictor of outcome, attributable in part to tumor edema. In this study, we removed the edematous signal to highlight the percentage of the involved volume of the CST. The combination of PIV and LTD index revealed a direct association with motor function decline. Our results suggested that an increase in volume in the edema area and decrease in LTD may reflect motor function decline (Figure 8).

Furthermore, the fiber tracts located in areas overlapping with peritumoral edema that only existed in NODDI-derived tractography (N-only condition) significantly increased and decreased in VF_*iso*_ and VF_*ic*_ when compared with the DN-only condition (Figure 9). In the DTI model, the FA index was calculated by a total signal in each voxel. Unlike DTI, the NODDI model separated the total signal into three compartments: VF_*ic*_, VF_*ec*_, and VF_*iso*_. However, in the calculation of NODDI’s ODI, only VF_*ic*_ and VF_*ec*_ were used (Zhang et al., 2012). While comparing the NODDI indices between DN and N-only conditions, we found that there were no significant differences in the VF_*ec*_, but there were in VF_*ic*_ and VF_*iso*_. The region with increasing isotropic volume fraction (increased water content of the surrounding tissue) causes the FA’s decrease and failure to reconstruct the CST in DTI tractography. This suggested that by excluding the isotropic volume fraction (edema effect), NODDI-derived tractography could reveal the fiber tracts located in areas overlapping with peritumoral edema. Based on the biophysical model, the increase in VF_*iso*_ may reflect the severity of vasogenic edema. Similarly, the decrease in VF_*ic*_ (neurite density index) may reflect focal neurologic deficits (neuron injury or axonal loss) and could also refer to the cognitive function decline (Kaal and Vecht, 2004; Parker et al., 2018). The edematous fiber tracts that only existed in DTI-derived tractography (D-only condition) found significant differences in the comparison between patients with and without motor function decline. However, as the D-only volume was relatively small (D-only volume = 31.45 ± 54.56 mm^3^), the estimation of the differences within these regions may come from the variability of the fiber orientation estimated by the DTI model.

### Limitations

Despite the good performance of the NODDI model in revealing the fiber tracts located in areas overlapping with peritumoral edema, our study has some limitations. First, our sample populations included a variety of tumor types. Previous studies have suggested that patients with glioma presumably had tumor-infiltrated edema, which was different from the pure vasogenic edema (Lu et al., 2003, 2004; Min et al., 2013). However, there were no significant differences found in the volume of edema with CST (DN-condition and N-only condition) in our dataset, which may suggest that patients in our dataset with edematous CST may not have infiltration by the tumor cell. The group comparison between vasogenic edema and edematous CST analysis can be found in Supplementary Material. Second, our MRI data were recruited from two different centers since these datasets were hard to obtain. Although the quantitative measurement that we used from the NODDI model, such as ODI, was normalized and calibrated from 10 healthy participants (five participants from each center), the variability from the different scanners still needed to be taken seriously. Third, our main purpose was to investigate the effect of edema on routinely employed tractography in clinical practice. The effect of crossing fiber in human white matter (Jeurissen et al., 2013) should be considered, and it would be necessary to utilize a model that focuses on solving crossing fiber-like constrained spherical deconvolution (CSD) (Tournier et al., 2008) in the future studies. Fourth, the NODDI protocol we used in this study is more time-consuming than the regular clinical DTI. The suggested NODDI protocol was encoded with two shells, 30 directions with 700 s/mm^2^ and 60 directions with 2,000 s/mm^2^ (Zhang et al., 2012), which involved approximately 15 min in our scanner with |*G*_*max*_|=45*mT*/m. The balance of the trade-off between acquisition time and imaging resolution was shifted toward lower imaging resolution to decrease the acquisition time. Thus, we used 2.5 mm^3^ as the isotropic voxel size, which is an acceptable protocol (Mohd Taib et al., 2017). Fifth, the optimal ODI threshold of 0.45 was only calibrated for the CST. The ODI thresholds were calibrated for the corpus callosum (0.478) and CST (0.467) in the study conducted by Figini et al. (2014), with values closer to our optimal values. However, the ODI threshold may differ while reconstructing other tracts. Finally, the correlation with intraoperative stimulation was absent. The changes of the post-operative CST examination in our study could be affected by multiple factors, especially the surgical approach. Future studies will require a larger sample size and direct cortical stimulation for validating the ability of NODDI-derived fiber tractography in the region of vasogenic edema in a more direct way.

## Conclusion

Our results demonstrated that NODDI-derived fiber tractography had a great potential to improve the reconstruction of fiber tracking through regions of vasogenic edema. Increasing the isotropic volume fraction or decreasing the intracellular volume fraction may cause the DTI tractography failure to reveal the fiber tracts located in areas with peritumoral edema. Moreover, the volume of edematous CST that was reconstructed by NODDI-derived tractography could provide an accurate microstructure index for clinical quantitative measurements and diagnostic performance. Therefore, this technique may help neurosurgeons to define a better and safe margin for tumor resection.

## Data Availability Statement

The raw data supporting the conclusions of this article will be made available by the authors, without undue reservation.

## Ethics Statement

The studies involving human participants were reviewed and approved by Institutional Review Board, Tri-Service General Hospital (TSGHIRB No. 1-102-05-109) Institutional Review Board, First Hospital of Jilin University (2017-465). The patients/participants provided their written informed consent to participate in this study. Written informed consent was obtained from the individual (s) for the publication of any potentially identifiable images or data included in this article.

## Author Contributions

SC, H-WK, and C-PL contributed to the conception and design of the study. XL and H-WK organized the database. SC, C-YEL, C-CH, Y-CK, K-TK, C-CHH, and C-YL provided the methodology support. SC wrote the first draft of the manuscript. SC, H-WK and C-PL wrote sections of the manuscript. YL and GZ provide the additional clinical suggestions during the revision. All authors contributed to manuscript revision, read, and approved the submitted version.

## Conflict of Interest

C-YEL was employed by GE Healthcare. The remaining authors declare that the research was conducted in the absence of any commercial or financial relationships that could be construed as a potential conflict of interest.

## Publisher’s Note

All claims expressed in this article are solely those of the authors and do not necessarily represent those of their affiliated organizations, or those of the publisher, the editors and the reviewers. Any product that may be evaluated in this article, or claim that may be made by its manufacturer, is not guaranteed or endorsed by the publisher.

## Footnotes

1
https://www.mrtrix.org/

2
https://fsl.fmrib.ox.ac.uk/fsl/fslwiki/

3
http://picsl.upenn.edu/ANTS

4
http://www.nitrc.org/projects/noddi_toolbox/

5
http://www.itksnap.org

6
http://dsi-studio.labsolver.org
