# Corrigendum: Exploring Peritumoral Neural Tracts by Using Neurite Orientation Dispersion and Density Imaging

**DOI:** 10.3389/fnins.2021.800009

**Published:** 2021-11-15

**Authors:** Shin Tai Chong, Xinrui Liu, Hung-Wen Kao, Chien-Yuan Eddy Lin, Chih-Chin Heather Hsu, Yi-Chia Kung, Kuan-Tsen Kuo, Chu-Chung Huang, Chun-Yi Zac Lo, Yunqian Li, Gang Zhao, Ching-Po Lin

**Affiliations:** ^1^Institute of Neuroscience, National Yang Ming Chiao Tung University, Hsinchu, Taiwan; ^2^Department of Neurosurgery, First Hospital of Jilin University, Changchun, China; ^3^Department of Medical Imaging, Hualien Tzu Chi Hospital, Buddhist Tzu Chi Medical Foundation, Hualien, Taiwan; ^4^Department of Radiology, School of Medicine, Tzu Chi University, Hualien, Taiwan; ^5^GE Healthcare, Taipei, Taiwan; ^6^Center for Geriatrics and Gerontology, Taipei Veterans General Hospital, Taipei, Taiwan; ^7^School of Psychology and Cognitive Science, Institute of Cognitive Neuroscience, East China Normal University, Shanghai, China; ^8^Institute of Science and Technology for Brain-Inspired Intelligence, Fudan University, Shanghai, China; ^9^Brain Research Center, National Yang Ming Chiao Tung University, Hsinchu, Taiwan

**Keywords:** neurite orientation dispersion and density imaging, diffusion tensor imaging, fiber tractography, vasogenic edema, brain tumor, neurosurgery

In the original article, there was an error. A typographical error was made for the IRB No. in the Ethics Statement.

A correction has been made to “*Ethics statement.”* The corrected statement is below.

The studies involving human participants were reviewed and approved by Institutional Review Board, Tri-Service General Hospital (TSGHIRB No. 1-102-05-109) Institutional Review Board, First Hospital of Jilin University (2017-465). The patients/participants provided their written informed consent to participate in this study. Written informed consent was obtained from the individual (s) for the publication of any potentially identifiable images or data included in this article.

The authors apologize for this error and state that this does not change the scientific conclusions of the article in any way. The original article has been updated.

## Publisher's Note

All claims expressed in this article are solely those of the authors and do not necessarily represent those of their affiliated organizations, or those of the publisher, the editors and the reviewers. Any product that may be evaluated in this article, or claim that may be made by its manufacturer, is not guaranteed or endorsed by the publisher.

