# Supplementary material for: Exploring Peritumoral Neural Tracts by Using Neurite Orientation Dispersion and Density Imaging
Source: Front Neurosci. 2021 Sep 27;15:702353. doi: 10.3389/fnins.2021.702353 (PMC8502884; doi:10.3389/fnins.2021.702353)
Supplement: Supplementary file 1 [file Data_Sheet_1.docx]

**Group comparison between vasogenic edema and edematous CST**

Previous studies suggested that patients with glioma presumably surrounded by tumor-infiltrated edema, which was different from the pure vasogenic edema (Lu et al., 2003;Lu et al., 2004;Min et al., 2013).Since our studies’ populations included various tumor types thus, we tested a group comparison between vasogenic edema and the edematous CST to endorse the feasibility of our analysis.

The patients with presumed purely vasogenic edema (meningiomas and metastatic lesion, n=13) were grouped and compared with patients presumably surrounded by tumor-infiltrated edema (low- and high-grade glioma, n=6). The DTI indies included fractional anisotropy (FA), mean diffusivity (MD), axial diffusivity (AD), and radius diffusivity (RD), and NODDI indices included intra-cellular volume fraction (VF_ic_), extracellular volume fraction (VF_ec_), and isotropic volume fraction (VF_iso_) were used to compare in the total volume of edema andthe regions of edema with CST (DN condition and N-only condition). Mann-Whitney U test was used for statistical comparison. All statistical analyses were analyzed using statistical software (SPSS, version 25; SPSS, Chicago, Ill). P-values less than 0.05 were considered statistically significant.

Comparing between the pure vasogenic edema and tumor-infiltrated edema in each group, mean MD (p<0.0047), AD (p<0.000), RD (p<0.0125), and VF_iso_ (p<0.0167) in the total volume of edema were found significant decrease in the tumor-infiltrated edema group. However, no significant difference was found in the region of edema with CST (DN condition and N-only condition). The comparison was summarized in Table S1.

Similar to previous studies, we found that the mean peritumoralMD of tumor-infiltrated edema was significantly lower than pure vasogenic edema. Therewere no significant differences in FA index(Lu et al., 2003;Lu et al., 2004). This significantly decreases pattern was also found in NODDIVF_iso_index. Which suggected that pure vasogenic edema reflects greater increased water content of the surrounding tissue.Moreover, the decreasing of FA (axonal disorganization contributing to FA decrease), and the increasing of VF_ic_ (more cellularity) in edema might be suggesting the tumor cell infiltrated. However, no significant differences were found in FA (p=0.2818) and VF_ic_ (p=0.0874).Furthermore, while only focusing on the region of edema with CST pass through (DN condition and N-only condition), no significant differences were found in each index.Our result suggested thatthe comparison between DN condition and N-only condition in our study might not be affected byvarious tumor types.

**Group comparison between patients with and without motor function decline.**

A total of 7 patients were reported with motor function decline. Twelve indices were used to determine the diagnostic performance for the patients’ motor function. The indices included: age, sex, LTD, edema volume, volume of D-only condition, volume of N-only condition, volume of DN condition, PIV of D-only condition, PIV of N-only condition, PIV of DN condition, volume of N-only condition weighted by LTD, and PIV of N-only condition weighted by LTD. The Wilcoxon Signed Ranks Test was conducted to evaluate the differences between patients with and without motor function decline. All statistic analyses were analyzed using statistical software (SPSS, version 25; SPSS, Chicago, III). P-values less than 0.05 were considered statistically significant.

We found that LTD (p<0.013), volume of D-only condition (p<0.024), PIV of D-only condition (p<0.017), volume of N-only condition (p<0.006), PIV of N-only condition (p<0.003), volume of N-only condition weighted by LTD (p<0.000), and PIV of N-only condition weighted by LTD (p<0.000) have significant differences between patient with and without motor function decline. The statistic details were summarized in Table S2.

Among these results, the PIV of N-only condition weighted by LTD had the best diagnostic performance for determining the presence of the patients’ motor function decline. The combining of PIV and LTD index revealed a direct association with motor function decline. Our results suggested that an increase in volume in the edema area and decrease in LTD may reflect motor function decline.

**Table S1. Group comparison between vasogenic edema and edematous CST.**

|  | **Edema (all)** | | | **Edema with CST** | | | | | |
| --- | --- | --- | --- | --- | --- | --- | --- | --- | --- |
|  |  |  |  | **Edema (DN-condition) (n=13)** | | | **Edema (N-only condition) (n=6)** | | |
|  | **Pure vasogenic edema** | **Tumor-infiltratededema** | **P-value** | **Pure vasogenic edema** | **Tumor-infiltratededema** | **P-value** | **Pure vasogenic edema** | **Tumor-infiltratededema** | **P-value** |
| **mean FA** | 0.1699±0.0322 | 0.1533±0.0131 | 0.2818 | 0.2108±0.1039 | 0.2577±0.0394 | 0.2065 | 0.1771±0.0407 | 0.1921±0.0378 | 0.3229 |
| **mean MD(×10^−3^mm^2^/s)** | 1.4862±0.1010 | 1.3660±0.0473 | **0.0047** | 1.0694±0.4837 | 1.2172±0.1235 | 0.8801 | 1.4671±0.1418 | 1.3405±0.1804 | 0.1518 |
| **mean AD(×10^−3^mm^2^/s)** | 1.7454±0.0881 | 1.5758±0.0646 | **0.0000** | 1.3573±0.6125 | 1.5509±0.1299 | 0.8801 | 1.7393±0.1358 | 1.6106±0.1956 | 0.2100 |
| **mean RD(×10^−3^mm^2^/s)** | 1.3566±0.1126 | 1.2612±0.0406 | **0.0125** | 0.9254±0.4223 | 1.0503±0.1253 | 1 | 1.3309±0.1521 | 1.2054±0.1754 | 0.1791 |
| **mean VF_ic_** | 0.1410±0.0245 | 0.1646±0.0258 | 0.0874 | 0.1815±0.0890 | 0.2267±0.0470 | 0.3607 | 0.1475±0.0474 | 0.1808±0.0616 | 0.2441 |
| **mean VF_ec_** | 0.5996±0.0531 | 0.6564±0.0647 | 0.1061 | 0.4838±0.2181 | 0.5903±0.0737 | 0.4076 | 0.5727±0.0515 | 0.6040±0.0850 | 0.4670 |
| **mean VF_iso_** | 0.2855±0.0598 | 0.2174±0.0486 | **0.0167** | 0.1808±0.0966 | 0.1830±0.0940 | 1 | 0.2798±0.0671 | 0.2152±0.0959 | 0.1791 |

**CST, corticospinal tract; FA, fractional anisotropy; MD, mean diffusivity; AD, axial diffusivity; RD, radius diffusivity; VF_ic_, intra-cellular volume fraction; VF_ec_, extra-cellular colume fraction; VF_iso_, isotropic volume fraction.**

**Table S2: Group comparisonbetween patients with and without motor function decline.**

|  | **Sex** | **Age** | **LTD** | **Edema volume** | **D-only volume** | **DN volume** | **N-only volume** | **N-only volume-LTD** | **D-only PIV** | **DN PIV** | **N-only PIV** | **N-only PIV-LTD** |
| --- | --- | --- | --- | --- | --- | --- | --- | --- | --- | --- | --- | --- |
| Mean | - | 52.08 | 19.52 | 16885 | 31.45 | 2377.71 | 827.03 | 87.80 | 0.0044 | 0.0555 | 0.1424 | 0.0168 |
| Std. | - | 12.18 | 15.02 | 8927 | 54.56 | 912.82 | 847.21 | 139.88 | 0.0067 | 0.0434 | 0.1407 | 0.0296 |
| Mann-Whitney U | 43 | 58.5 | 20.5 | 37 | 24 | 53 | 16 | 2 | 22 | 38 | 13 | 0 |
| Wilcoxon W | 196 | 211.5 | 48.5 | 65 | 52 | 81 | 169 | 155 | 50 | 66 | 166 | 153 |
| Z | -1.249 | -0.064 | -2.478 | -1.429 | -2.269 | -0.413 | -2.763 | -3.652 | -2.384 | -1.366 | -2.953 | -3.779 |
| Asymp. Sig. | 0.212 | 0.951 | **0.013** | 0.153 | **0.024** | 0.680 | **0.006** | **0.000** | **0.017** | 0.172 | **0.003** | **0.000** |

**PIV, percentage of involved volume; LTD, lesion-to-tract distance; Std, standard deviation; Asymp. Sig., asymptotic significance.**

**
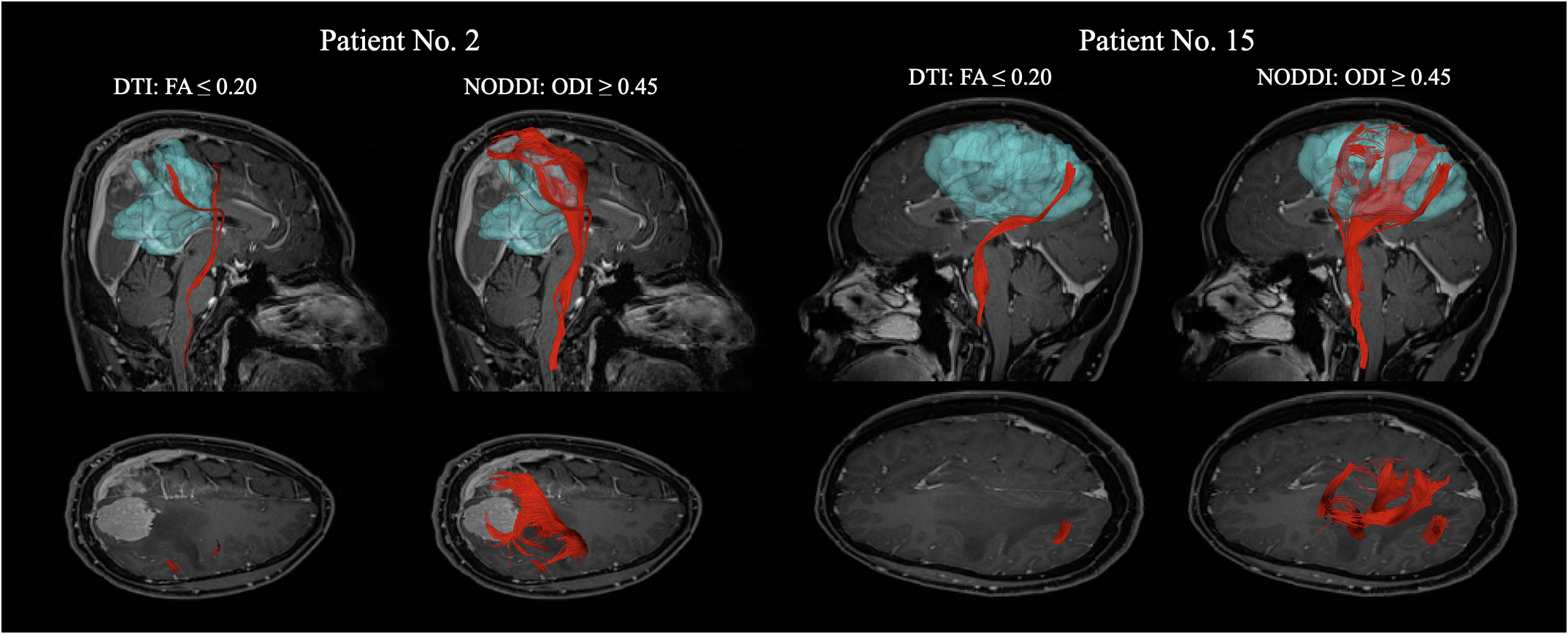
**

Figure S1.

DTI failed to reconstruct any of the CST in the regions with vasogenic edema.

Patients No. 2, a 42-year-old man with a meningioma (WHO grade I) in the right parietal lobe(left) and patient No. 15, a 51-year-old woman with lung metastatic in the left parietal lobe (right). Two patients failed to reconstruct any of the CST (red) in the regions with vasogenic edema (light blue) while using DTI tractography with standard termination criteria.

DTI, diffusion tensor imaging; NODDI, neurite orientation dispersion and density imaging; CST, corticospinal tract; FA, fractional anisotropy; ODI, orientation dispersion index and WHO, World Health Organization.
